# Supplementary material for: The Prognostic Significance of FKBP1A and Its Related Immune Infiltration in Liver Hepatocellular Carcinoma
Source: Int J Mol Sci. 2022 Oct 24;23(21):12797. doi: 10.3390/ijms232112797 (PMC9659304; doi:10.3390/ijms232112797)
Supplement: Supplementary file 1 [file ijms-23-12797-s001.zip › ijms-1895420-supplementary.pdf]

Supplementary Table S1. **Expression levels of *FKBP1A* in HCCDB database**

Dataset	P-value	Type	Nums	Mean	STD	IQR
**HCCDB1**	0.000001070	HCC	100	11.34	0.3667	0.4914
		Adjacent	97	11.10	0.3168	0.3249
**HCCDB3**	0.0001361	HCC	268	7.896	1.791	2.210
		Adjacent	243	7.311	1.644	1.835
		Cirrhotic	40	10.23	1.665	1.871
		Healthy	6	6.262	0.2864	0.4250
**HCCDB4**	4.570e-31	HCC	240	8.183	0.6289	0.9086
		Adjacent	193	7.536	0.4386	0.5385
**HCCDB6**	1.390e-24	HCC	225	6.447	0.6841	0.9070
		Adjacent	220	5.824	0.5076	0.6724
**HCCDB7**	0.03196	HCC	80	14.12	0.5324	0.7195
		Adjacent	82	13.93	0.5670	0.8197
**HCCDB11**	0.06864	HCC	88	8.013	0.5790	0.5815
		Adjacent	48	8.260	0.8235	0.7188
**HCCDB12**	0.00001900	HCC	81	7.966	0.4326	0.6260
		Adjacent	80	7.692	0.3512	0.4174
**HCCDB13**	0.2174	HCC	228	5.860	0.9144	1.029
		Adjacent	168	5.726	0.7040	0.9128
**HCCDB15**	9.180e-18	HCC	351	11.84	0.5789	0.7500
		Adjacent	49	11.33	0.2762	0.4200
**HCCDB16**	9.020e-9	HCC	60	9.202	0.3315	0.3739
		Adjacent	60	8.857	0.2765	0.3266
**HCCDB17**	3.500e-10	HCC	115	9.109	0.4034	0.6050
		Adjacent	52	8.661	0.3780	0.4300
**HCCDB18**	3.000e-39	HCC	212	6.092	0.5949	0.7875
		Adjacent	177	5.292	0.4774	0.4800

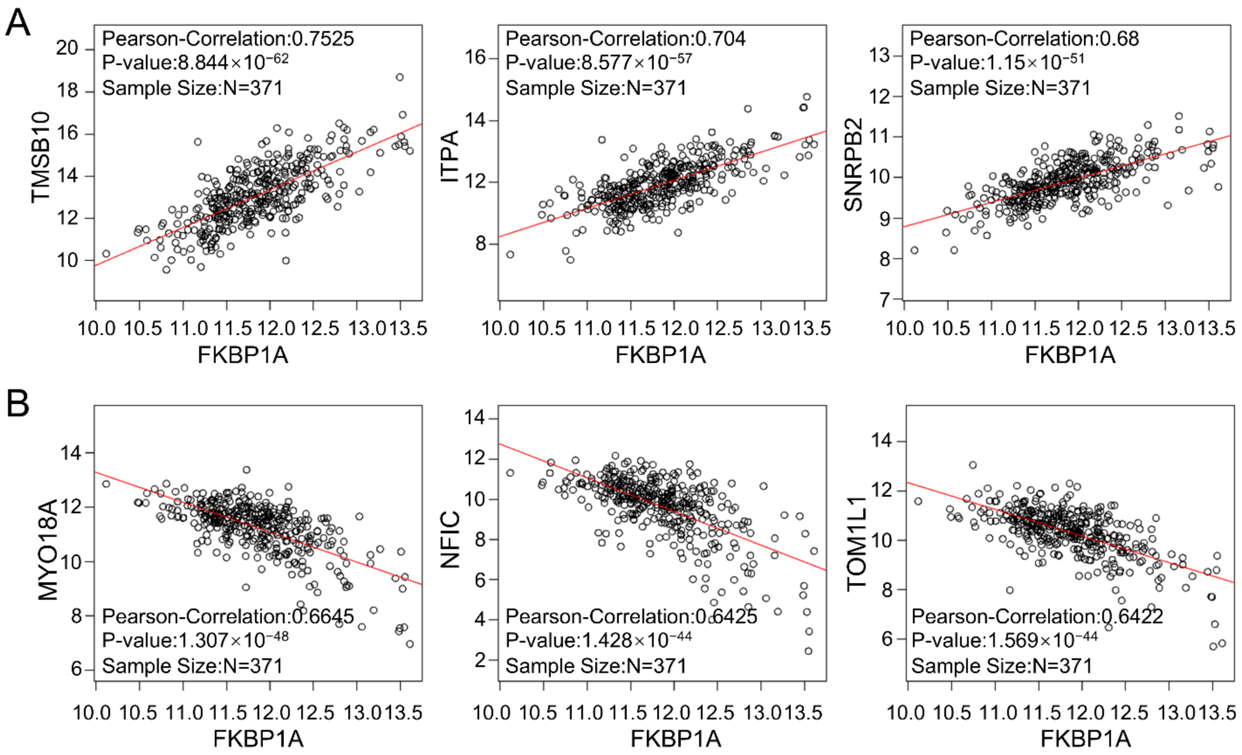

Supplementary Figure S1. The top3 co-expression genes with *FKBP1A* were analyzed in LIHC from the LinkedOmics database. (A-B) The scatter plot shows the Pearson correlation of *FKBP1A* expression with the top3 positively correlated genes (A) and negatively correlated genes (B) in LIHC.

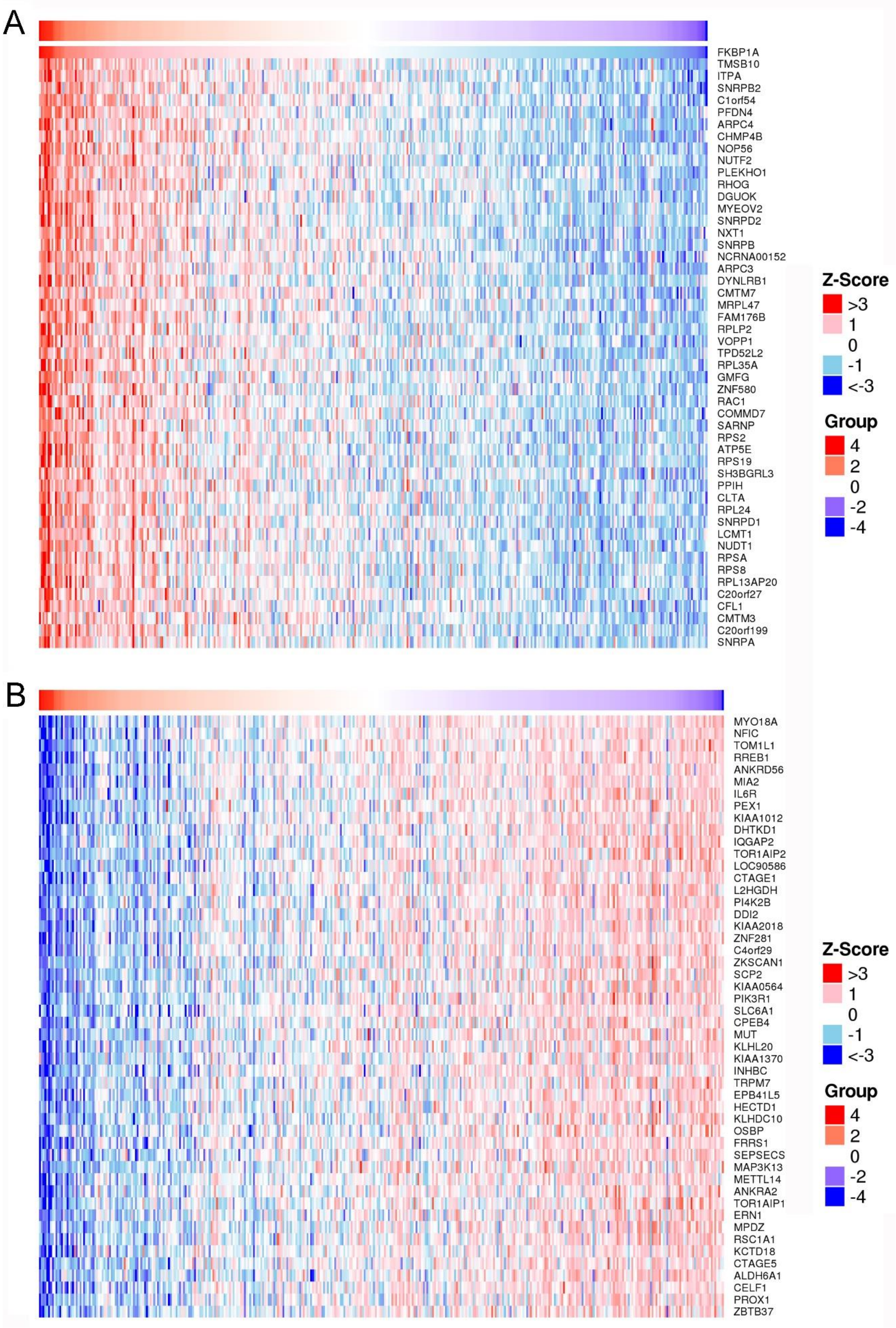
A
FKBP1A
TMSB10
ITPA
SNRPB2
C1orf54
PFDN4
ARPC4
CHMP4B
NOP56
NUTF2
PLEKHO1
RHOG
DGUOK
MYEOV2
SNRPD2
NXT1
SNRPB
NCRNA00152
ARPC3
DYNLRB1
CMTM7
MRPL47
FAM176B
RPLP2
VOPP1
TPD52L2
RPL35A
GMFG
ZNF580
RAC1
COMMD7
SARNP
RPS2
ATP5E
RPS19
SH3BGRL3
PPIH
CLTA
RPL24
SNRPD1
LCMT1
NUDT1
RPSA
RPS8
RPL13AP20
C20orf27
CFL1
CMTM3
C20orf199
SNRPA
Z-Score
>3
1
0
-1
<-3
Group
4
2
0
-2
-4
B
MYO18A
NFIC
TOM1L1
RREB1
ANKRD56
MIA2
IL6R
PEX1
KIAA1012
DHTKD1
IQGAP2
TOR1AIP2
LOC90586
CTAGE1
L2HGDH
PI4K2B
DDI2
KIAA2018
ZNF281
C4orf29
ZKSCAN1
SCP2
KIAA0564
PIK3R1
SLC6A1
CPEB4
MUT
KLHL20
KIAA1370
INHBC
TRPM7
EPB41L5
HECTD1
KLHDC10
OSBP
FRRS1
SEPSECS
MAP3K13
METTL14
ANKRA2
TOR1AIP1
ERN1
MPDZ
RSC1A1
KCTD18
CTAGE5
ALDH6A1
CELF1
PROX1
ZBTB37
Z-Score
>3
1
0
-1
<-3
Group
4
2
0
-2
-4

Supplementary Figure S2. Top 50 genes related to *FKBP1A* in LIHC are shown in heatmaps from the LinkedOmics database. (A-B) Heat maps showing top 50 genes positively (A) and negatively (B) correlated with *FKBP1A*. Red indicates positively correlated genes and blue indicates negatively correlated genes.

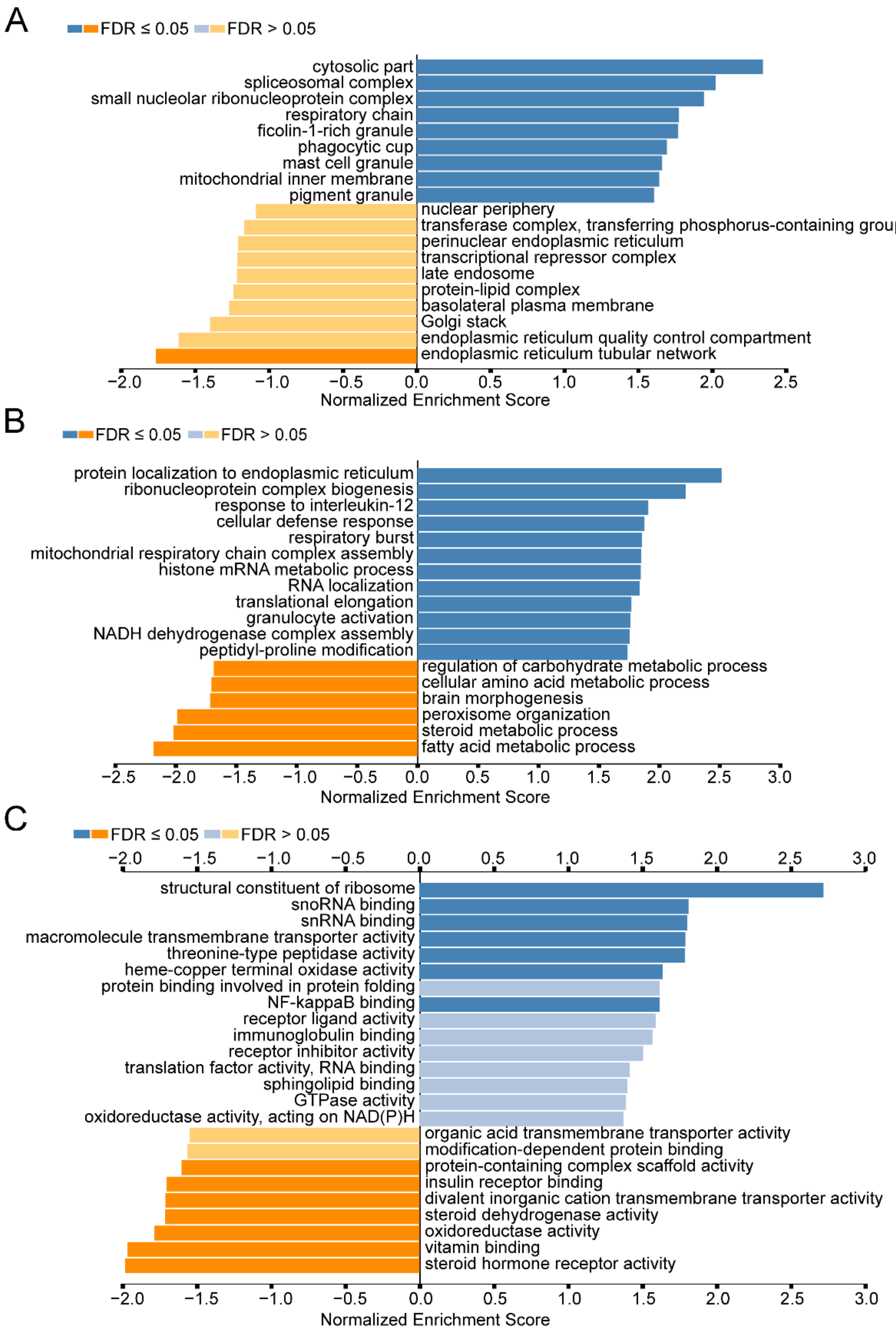

Supplementary Figure S3. The significantly enriched GO annotations of genes co-expressed with *FKBP1A* were analyzed using gene set enrichment analysis by LinkedOmics database. (A) Cellular components. (B) Biological processes. (C) Molecular functions. FDR, false discovery rate. The x-axis represents the normalized enrichment score, and the y-axis represents the term of GO. GO, Gene Ontology; FDR, false discovery rate.

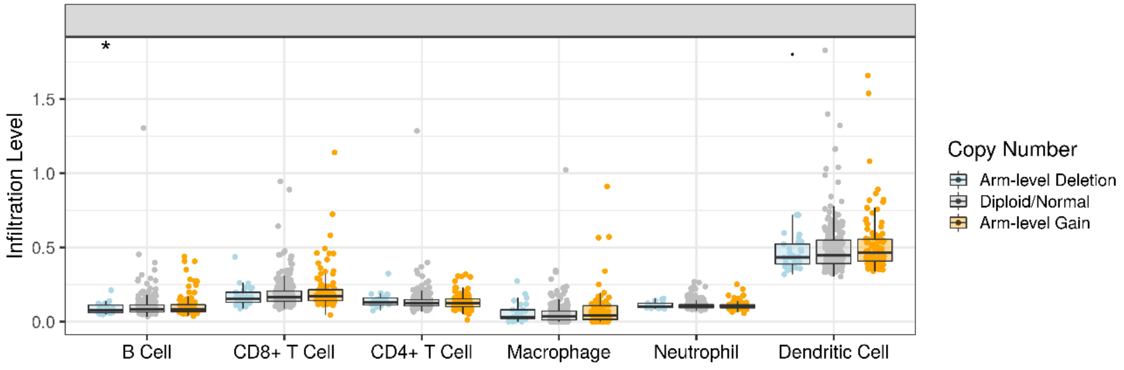

Supplementary Figure S4. The infiltration level of various immune cells under somatic copy number alterations of *FKBP1A* in LIHC. *$p < 0.05$.

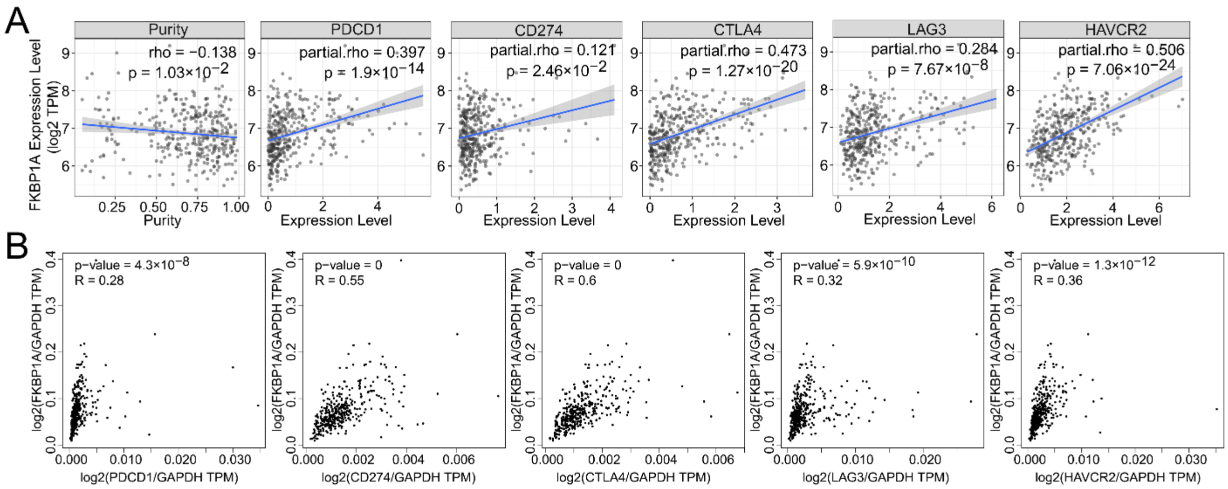

Supplementary Figure S5. Correlation of *FKBP1A* expression with *PD-1*, *PD-L1*, *CTLA-4*, *LAG3* and *HAVCR2* expression in LIHC. (A) Spearman correlation of *FKBP1A* with expression of *PD-1*, *PD-L1*, *CTLA-4*, *LAG3* and *HAVCR2* in LIHC adjusted by tumor purity using TIMER2.0. (B) The expression correlation of *FKBP1A* with expression of *PD-1*, *PD-L1*, *CTLA-4*, *LAG3* and *HAVCR2* in LIHC determined by GEPIA database.
